# Reflecting on the quality of a methodologically pluralist evaluation of a large-scale Indigenous health research collaboration in Australia

**DOI:** 10.1136/bmjgh-2023-014433

**Published:** 2024-08-03

**Authors:** Jodie Bailie, Veronica Matthews, Alison Frances Laycock, Kathleen Conte, Lynette Feeney, Roxanne Bainbridge

**Affiliations:** 1 University Centre for Rural Health, The University of Sydney, Lismore, New South Wales, Australia; 2 Centre for Disability Research and Policy, The University of Sydney, Sydney, New South Wales, Australia; 3 Homelessness Research and Action Collaborative, Portland State University, Portland, Oregon, USA; 4 Poche Centre for Indigenous Health, The University of Queensland, Brisbane, Queensland, Australia

**Keywords:** Health services research, Health systems evaluation, Qualitative study

## Abstract

**Background:**

Indigenous communities worldwide lead calls for all evaluations of research, programmes and policies affecting their communities to reflect the values, priorities and perspectives of the Indigenous peoples and communities involved. Tools, such as the Quality Appraisal Tool (QAT), are available to assess research quality through an Indigenous cultural lens. Good evaluation requires that evaluation efforts be evaluated. We found that critical reflection on the quality of evaluations from an Indigenous perspective is largely absent from the published literature. To ensure that we strive for quality in evaluation as determined by Indigenous people with whom we work, we examined the quality of our own evaluation of an Indigenous health research collaboration by conducting a reflexive dialogue.

**Methods:**

The QAT was used to assess our evaluation according to Indigenous health research principles. Our qualitative study used analytical coautoethnography to generate data through a series of reflexive dialogue sessions with Indigenous and non-Indigenous members of the research collaboration, using the QAT criteria as discussion prompts. Our ideas and reflections were compared and contrasted through a collaborative and iterative writing process, multiple review cycles and discussions.

**Results:**

We documented our findings against the QAT framework. We found examples that each QAT principle had, to some extent, been adhered to, but constantly needed to assess whether the principles were fully achieved to our satisfaction. Strengths of the evaluation included being adaptable and responsive to emerging issues for the research collaboration, while areas for improvement included more Indigenous leadership of, and involvement in, evaluation.

**Conclusions:**

Although reflexive evaluation practice is not always comfortable, it does provide an opportunity to generate insights for improvement. Reflecting as we did—in a partnership between Indigenous and non-Indigenous colleagues—enabled deeper insights and meaning. We anticipate that our process models how other research in Indigenous contexts might better advance ethical, quality Indigenous research through working in collaboration with Indigenous researchers and communities.

**WHAT IS ALREADY KNOWN ON THIS TOPIC**

- Indigenous communities worldwide are leading calls for all evaluations of research affecting Indigenous peoples to reflect the values, priorities and perspectives of the Indigenous peoples and communities involved.
- To date, critical reflection on the quality of evaluations from an Indigenous viewpoint is largely absent from the published literature.

**WHAT THIS STUDY ADDS**

- A reflexive dialogue that examines the quality of an evaluation of an Indigenous health research collaboration using an Indigenous Quality Appraisal Tool.
- Strengths of the evaluation included its adaptability and responsiveness to emerging issues within the research collaboration.
- Areas for improvement included the need for more Indigenous leadership and involvement in the evaluation.

**HOW THIS STUDY MIGHT AFFECT RESEARCH, PRACTICE OR POLICY**

- Our reflexive evaluation approach models how other researchers and evaluators working with Indigenous people might improve their ethics of practice and better advance the quality of collaborative leadership.

## Background

 Programme evaluation can contribute to improving health outcomes for Aboriginal and Torres Strait Islander people (Aboriginal and Torres Strait Islander peoples are hereafter referred to respectfully as Indigenous Australians, acknowledging cultural and historical diversity) by informing the design and implementation of research, programmes and policy and generating new knowledge on their effectiveness and impact. It can also strengthen the evidence base, which then informs future interventions.^1^ However, concerns have been raised that the evaluations of programmes addressing Indigenous health does not always deliver these benefits.^2 3^

Good evaluation requires that evaluation efforts themselves be evaluated.^4^,^6^Thus, there are calls for evaluations to incorporate the values and perspectives of the Indigenous people involved more effectively.^1^,^37^ Meaningful engagement with Indigenous people must occur early through codesign and be sustained throughout the evaluation to coproduce actionable knowledge, then cocreate interventions, programmes and policies.^3 8 9 11 12^

Central to improving the quality of evaluations is the need for researchers and other stakeholders to practise reflexivity, in which they examine their own beliefs and judgements and how these impact on engagement with communities and the coproduction of knowledge.^713 15^,^17^ Furthermore, Liwanag and Rhule^17^ argue that reflexivity must include a self-reflection process, and also additional elements of reflexive dialogues with peers and others who might offer alternative perspectives and a process for any resulting insights to lead to action. Despite reflexivity being identified as a hallmark of good evaluation for decades, there is still a dearth of peer-reviewed literature reflecting on the quality of an evaluation from an Indigenous perspective.^18^

Despite the continued dominance of Western evaluation frameworks, there has been an international increase in Indigenous-led evaluations and a growing trend of cross-cultural collaborations and codesign. This includes partnerships between Indigenous and non-Indigenous groups, as well as the development of culturally responsive evaluation resources and guiding principles.^18^,^21^ Within Australia, there are, a number of principles to guide and shape research and evaluation involving Indigenous people, such as those recommended by the National Health and Medical Research Council (NHMRC),^22^ the Australian Evaluation Society First Nations Cultural Safety Framework^23^ and research checklists such as the Consolidated Criteria for Strengthening Reporting of Health Research involving Indigenous Peoples (CONSIDER) statement. Developed by Harfield *et al*, the Aboriginal and Torres Strait Islander Quality Appraisal Tool^11 24^ assesses the quality of research through an Indigenous cultural lens (box 1). Online supplemental file 1 describes each QAT domain in detail.

Box 1 **Domains of the Quality Appraisal Tool for Indigenous research^11^**

1. Did the research respond to a need or priority determined by the community?
2. Was community consultation and engagement appropriately inclusive?
3. Did the research have Indigenous leadership?
4. Did the research have Indigenous governance?
5. Were local community protocols respected and followed?
6. Did the researchers negotiate agreements in regard to rights of access to Indigenous peoples’ *existin**g* intellectual and cultural property?
7. Did the researchers negotiate agreements to protect Indigenous peoples’ ownership of intellectual and cultural property *created* through the evaluation?
8. Did Indigenous peoples and communities have control over the collection and management of research materials?
9. Was the research guided by an Indigenous research paradigm?
10. Did the research take a strengths-based approach, acknowledging and moving beyond practices that have harmed Indigenous peoples in the past?
11. Did the researchers plan and translate the findings into sustainable changes in policy and/or practice?
12. Did the research benefit participants and Indigenous communities?
13. Did the research demonstrate capacity strengthening for Indigenous individuals?
14. Did everyone involved in the research have opportunities to learn from one another?

The QAT was designed for a broad application that included educating researchers about how to conduct respectful, high-quality research with Indigenous peoples and communities, thereby ultimately increasing the relevance and benefit of health research to these communities. All 14 questions in the QAT were thus informed by Indigenous research values and ethics, and encompass the following domains: setting appropriate research questions; community engagement and consultation; research leadership and governance; community protocols; intellectual and cultural property rights; the collection and management of research material; Indigenous research paradigms; a strength-based approach to research; the translation of findings into policy and practice; benefits to participants and communities involved and capacity strengthening and two-way learning.^11^ Harfield *et al*^11^ call for application of the QAT to strengthen its utility and ongoing refinements.

In this paper, we used the QAT to assess the quality of an evaluation of a national Indigenous health research collaboration—the Centre for Research Excellence in Integrated Quality Improvement (CRE-IQI) (box 2).^25^,^27^ Decolonising evaluation through collaborative efforts resets power dynamics by centring Indigenous voices, perspectives and priorities in the evaluation process. This approach challenges historical hierarchical structures in research and evaluation, altering colonial legacies. By doing so, we inform our ongoing practice as evaluators and enable others to learn from our evaluation experience, thereby improving future evaluation practices and contributing to better understanding and foregrounding of Indigenous voices.

Box 2 **Centre for Research Excellence in Integrated Quality Improvement**

Indigenous Australians demonstrate cultural resilience and adaptability, yet they continue to experience poorer health outcomes and shorter life expectancy compared with other Australians.^43^ These disparities stem from the enduring legacy of colonisation, including land dispossession, displacement, disempowerment, social and economic exclusion and persistent racism. Centuries of government paternalism and neglect further exacerbate these challenges, which Indigenous Australians continue to confront and work to redress.^44 45^

In a bid to improve Indigenous health, Australia’s National Health and Medical Research Council (NHMRC) funded the Centre for Research Excellence in Integrated Quality Improvement (CRE-IQI) from November 2014 to November 2019 to conduct research into the strengthening of primary healthcare (PHC) systems using continuous quality improvement (CQI).^25 46^ Building on more than two decades of participatory CQI research and development with Indigenous health services and communities, the CRE-IQI brought together Indigenous community-controlled and government-managed PHC centres with research institutions, government health departments and key regional support organisations (eg, health councils) to strengthen and embed system-wide CQI.^32^

The CRE-IQI collaboratively developed and refined both research priorities to address key stakeholder needs, and a set of principles to govern practice.^33^ It also encouraged new collaborations through the sharing of information, open seed-funding calls to develop projects and promoting collaborative research. The CRE-IQI held biannual face-to-face meetings, with research masterclasses conducted in association with the meetings, and online monthly research capacity building seminars. It also provided support for teams to pursue collaborative research and develop capacity, with numerous interlinked research projects and capacity-strengthening activities.^46 47^ Further details about how the CRE-IQI operated, descriptions of the research programmes and its research findings are published elsewhere.^27 46 47^

## Methods

### An evaluation of the Centre for Research Excellence in Integrated Quality Improvement

One of the primary research aims of the CRE-IQI was to evaluate the formation, functioning and outcomes of the CRE-IQI. The evaluation of the CRE-IQI was conceptualised as a developmental evaluation and implemented from the commencement of the CRE-IQI.^27^,^29^ Developmental evaluation embraces emergent and participatory approaches and, as such, is congruent with calls by Indigenous scholars for a systems science approach to address complex issues.^30^ Ensuring that our evaluation findings were useful was paramount, particularly because many of the end-users were participants in the CRE-IQI.^27^

**Figure 1:**
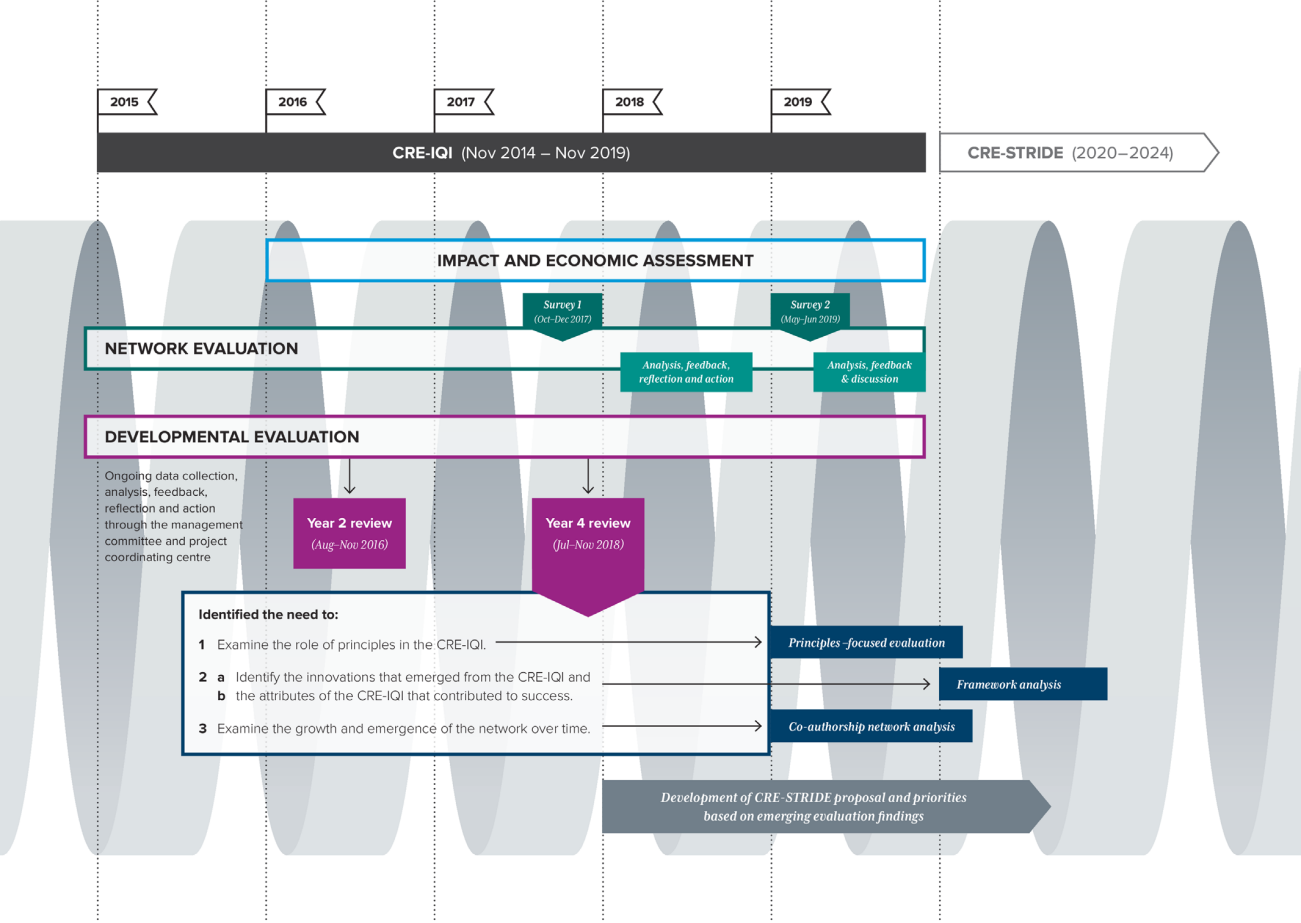
Key elements of the evaluation design of the CRE-IQI. (1) The spiral represents the interconnected elements of the evaluation design, incorporating processes for ongoing analysis, feedback and action. (2) The initial evaluation plan comprised an evaluation of impact and economics, network evaluation and developmental evaluation. (3) In keeping with the principles of developmental evaluation, over time we identified emerging issues that required different evaluation approaches, such as principles-focused evaluation, coauthorship network analysis and framework analysis. (4) CRE-IQI, Centre for Research Excellence in Integrated Quality Improvement; CRE-STRIDE, Centre for Research Excellence in Strengthening Systems for Indigenous Healthcare Equity.

Within this overarching framework of developmental evaluation,^26^ we implemented a methodologically pluralist evaluation design^27^ that, in its simplest form, denotes a diversity of methods. These included a developmental evaluation,^26^ social network analysis,^31^ coauthorship network analysis,^32^ principles-focused evaluation,^33^ framework analysis^34^ and an impact and economic evaluation.^35^ Several publications offer more details on the background, rationale, methods and findings for each evaluative approach or method used.^2627 31^,^35^ Some approaches and methods emerged throughout the lifecycle of the evaluation in response to evaluative feedback and discussions among the evaluation team—in particular, the principles-focused evaluation^33^ and the coauthorship network analysis.^32^ Importantly, the overarching developmental evaluation emphasised and embedded ongoing continuous reflection and learning across all of the methods used to evaluate the CRE-IQI. Figure 1 outlines the key elements of the CRE-IQI evaluation.

The effective conduct of the developmental evaluation was one of the primary responsibilities of the CRE-IQI Research Fellow (Evaluation) (JB). The Fellow was guided by an Evaluation Working Group (EWG) that met monthly via teleconference and every 6 months face-to-face. Key tasks of the EWG included designing and coordinating evaluative activities, along with the early analysis and interpretation of findings. The EWG, chaired by an Indigenous researcher/evaluator (RB), comprised researchers with specific evaluation skills and responsibilities within the CRE-IQI. Evaluation was also a standing item at the CRE-IQI bimonthly management committee meetings and 6 monthly biannual meetings with broader stakeholders.

### Study design

For this study, we adopted a critical theory perspective that posits research is never truly value-free and should only be conducted with an expressed goal of social change.^36^ To achieve this, we chose an analytic coautoethnography approach^37^ to collect and interpret our reflections using the QAT to guide a reflexive dialogue^17^ between Indigenous and non-Indigenous authors. Analytical coautoethnography is ethnographic work ‘in which the researcher: (1) is a full member in the research group or setting; (2) is visible as a member in the researcher’s published texts and (3) is committed to an analytic research agenda focused on improving theoretical understandings of broader social phenomena’^37^ (p375). The authors of this paper met all three of these criteria.

Coautoethnography met our methodological needs for three key reasons.^38^ First, it allowed us, as participant–researchers, to explore ourselves in the presence of others, fostering a collective understanding of our shared experiences. Second, coautoethnography stands as an empowering approach, lending itself to allowing agency and voice to shape the research narrative through shared experiences and dialogue. Third, coautoethnography aligns effectively with Indigenous research aims by critically examining the politics of representation, particularly regarding voice and its authenticity.

Reporting of our study was guided by the Standards for Reporting Qualitative Research (SRQR).^39^

### Locating the authors of this manuscript

We are a group of Indigenous (VM, RB and LF) and non-Indigenous (JB, AL and KC) researchers and evaluators who worked together on the CRE-IQI. The reflexive dialogue we undertook was sparked by conversations with several CRE-IQI members about the need to appraise the evaluation against established criteria for quality Indigenous health research, and to document and share key learnings to inform further evaluative work. All six authors are female with a long-standing history of working together in various capacities and a deep commitment to improving the health outcomes of Indigenous people. We were involved in the CRE-IQI in various ways, as follows.

*Aboriginal authors*: RB is a professor from the Gunggari/Kunja nations, and was chair of the EWG, a CRE-IQI chief investigator and coauthor on manuscripts relating to the CRE-IQI evaluation. VM is an associate professor from the Quandamooka community, and was a CRE-IQI research fellow, participant in the evaluation and coauthor of related manuscripts. LF is a health services researcher and evaluator from the Yankunytjatjara and Warumungu-Warlpiri nations and was a CRE-IQI chief investigator with a long-standing history of working with CRE-IQI research collaborators.

*Non-Indigenous authors*: JB was a CRE-IQI research fellow, embedded evaluator leading the CRE-IQI developmental evaluation, CRE-IQI project manager and a member of the EWG. AL was a CRE-IQI research fellow and member of the EWG, and a coauthor of manuscripts related to the CRE-IQI evaluation. KC was a CRE-IQI research fellow and a coauthor of several evaluation manuscripts.

### Data collection

The Indigenous authors (VM, RB and LF) and non-Indigenous authors (JB, AL and KC) met separately, via the online Zoom platform, to give a critical appraisal of the CRE-IQI evaluation based on the 14 domains in the QAT (box 1).^11^

Through a reflexive dialogue, both groups separately discussed and documented experiences, observations and knowledge of the CRE-IQI evaluation. Initially, we formed separate groups as an attempt to mitigate power imbalances, aiming to provide a safe space for the comprehensive development, presentation and discussion of perspectives. All authors had access to a QAT companion document which outlines examples of good practice to consider when assessing projects against the tool.^24^

Both groups used a consensus process to assess whether there was adequate evidence of adherence to the domains identified in the QAT. Using the QAT’s criterion scale of ‘Yes’, ‘Partially’, ‘No’ or ‘Unclear’ the two groups separately scored each domain and provided a written justification for their scoring. Because the questions in the QAT were phrased in a dichotomous way we rephrased them to suit our aim of a reflective process, while still maintaining their intent. For example, the QAT asks, ‘Did the research respond to a need or priority determined by the community’, which was rephrased as ‘To what extent did the evaluation respond to a need or priority determined by the community’ (online supplemental file 2). Because the scoring system did not work as well when the questions were reframed, we drifted to a more qualitative assessment. This process was iterative and enabled rich conversations, with both groups reconvening as necessary to review and refine their responses. Online supplemental file 2 contains the two groups initial perspectives. There were discussions about what the questions meant in the context of the *evaluation* of the CRE-IQI rather than the CRE-IQI as a whole or the individual research projects it spawned.

### Data analysis

Both sets of reflections from the Indigenous and the non-Indigenous authors were shared among all coauthors. Despite our efforts to focus on the *evaluation* of the CRE-IQI, at times our responses were more about the CRE-IQI collaboration as a whole and specific CRE-IQI research. To address this issue, we held several meetings to refine our responses, and to compare and contrast the findings.

Our ideas and reflections were refined through a collaborative and iterative writing process, involving multiple review cycles and discussions within the two groups and among all coauthors. Finally, using descriptions of the domains provided by Harfield *et al*,^11^ all authors checked that the findings were consistent with their own perceptions and understanding based on their experience as a CRE-IQI member and as authors of evaluation manuscripts and reports.

During analysis, it became apparent that the domains of quality espoused in the QAT were not independent and that some findings were relevant to more than one domain. Therefore, we describe findings according to their predominant domain and most important influence, and also collapse some domains to avoid repetition. To ensure we retain the voice and perspectives of Indigenous authors, we present quotes from Indigenous authors’ responses to the QAT questions in italics.

### Patient and public involvement

This research did not have patient or public involvement.

## Findings

### Responding to a need or priority determined by Indigenous community, and community consultation and engagement

An evaluation of the CRE-IQI was one of the five research aims defined in the initial funding proposal, and dedicated funding and leadership were committed to it. As most of our coauthor team had not been involved in the conceptualisation of the CRE-IQI nor the development of the funding proposal, we were unsure of the level of input by Indigenous people in determining the CRE-IQI research aims. However, we were aware that the CRE-IQI proposal built on almost two decades of collaborative research between researchers and Indigenous health services,^32^ and that evidence of community consultation was required as a condition of grant funding. There were also a number of Indigenous investigators and organisations named on the CRE-IQI grant, and CQI (a focus of the grant proposal) was identified as a high priority for Indigenous PHC services with the imminent release of the National Framework for CQI in Indigenous PHC.^40^

Specifically for the evaluation of the CRE-IQI, the Indigenous community voice was heard through Indigenous people who participated in the CRE-IQI; this included Indigenous researchers, policy makers and health service staff. However, during the reflexive dialogue, it became evident that our two groups differed in their definition and understanding of the ‘community voice’. Non-Indigenous authors perceived that in addition to Indigenous people, we heard community voice through representatives from Indigenous community-controlled organisations and Indigenous peak bodies, whereas the Indigenous authors maintained Indigenous voice is only from Indigenous people, not through proxy.

### Indigenous leadership and governance

The CRE-IQI was guided by a set of overarching principles,^33^ several of which advocated for Indigenous leadership and direction in all stages of research and evaluation, and an ‘all teach, all learn’ framework^41^ that emphasised and valued mutual learning. Because the evaluation was embedded within the CRE-IQI, it was guided by these principles and framework.

While the EWG was chaired by an Indigenous researcher and evaluator (RB), a relatively small proportion of Indigenous people were initially engaged in the leadership and governance of the CRE-IQI overall. During this reflexive dialogue, Indigenous authors described the effect of having limited Indigenous leadership, and the challenges that can arise when endeavouring to influence change: *You feel the weight of being the lone voice, feeling you had little influence and your voice is lost* (Indigenous coauthor)

In response to evaluative feedback and subsequent focused strategies, leadership and participation by Indigenous people increased over time. For example, targeted invitations, engaging agendas and financial support helped to strengthen the representation of Indigenous people at the CRE-IQI’s biannual meetings, where our evaluation plans and interim analysis of evaluation findings were workshopped.

### Aboriginal and Torres Strait Islander protocols respected and followed

The CRE-IQI used collaborative processes to develop principles of practice^33^ and an ‘all-teach, all-learn’ capacity-strengthening approach,^41^ both of which were intended to inform and guide any CRE-IQI activity, including the evaluation. Developmental evaluation methodology also emphasises mutual learning and respect for all contributions, so the ‘all-teach, all-learn’ approach was revisited, and reflected and built on throughout the evaluation. As a result, partnership and coleadership arrangements between Indigenous and non-Indigenous members of the collaboration were over time further developed. For example, all CRE-IQI seed funding for research projects were required to be either Indigenous-led or co-led.

### Agreements to protect existing and created intellectual and cultural property

For this QAT domain, we reflected on how the evaluation was primarily concerned with collecting and using data from CRE-IQI collaborators for the purpose of refining the ongoing operation of the collaboration. We considered how jointly created evaluative data were shared through the presentation of early findings to the CRE-IQI, and member checking and participatory analysis processes. Because the evaluation research involved many organisations and people, we considered the ownership of intellectual property to be dispersed as the collected evaluative data were used to inform ongoing cycles of reflection and action within the CRE-IQI collaboration.

We also discussed the importance placed by the CRE-IQI on ensuring that all evaluation outputs acknowledged the contributions of Indigenous people, evaluators and participants in the generation of new knowledge. For example, Indigenous authorship was mandated on all evaluation outputs, while author information on our peer-reviewed manuscripts included statements of the authors’ positionality, including their Indigenous status. We viewed these strategies as examples of how the CRE-IQI continuously sought to improve its commitment to Indigenous engagement and ownership. However, this domain of the QAT prompted reflection by the coauthors on the shortcomings of some academic institutions in honouring and protecting both data sovereignty and Indigenous intellectual and cultural property, as well as on the positive developments in this area.

### Control over the collection and management of evaluation materials

Both Indigenous and non-Indigenous CRE-IQI members participated in decisions on how information arising from the evaluation was to be disseminated and used to inform the ongoing operation of the CRE-IQI, with Indigenous input in these processes increasing over time. We increased Indigenous input by implementing purposeful strategies. This involved supporting Indigenous leadership and participation in CRE-IQI related meetings and providing safe spaces. During the research collaboration meetings, we presented early evaluation findings to all attendees. However, Indigenous people who participated in the evaluation had limited control over data collection and management. This was mainly because the management and storage of data collected as part of the evaluation was regulated by university standards and policies, which require data to be stored in secure University locations and be archived and destroyed within a specified time after project completion. These requirements were outlined in the ethics-approved participant information sheet, and as part of the informed consent processes specific to the evaluation.

### Indigenous research paradigms

The evaluation methodologies all came from Western research paradigms, so at times proved challenging to apply in an Indigenous context. As an example, the measurement indicators for the economic and impact evaluation^35^ needed to be, and were, modified to incorporate values and meaning for Indigenous people: *If this doesn’t occur, the evaluation won’t be relevant … or make sense for our mob* (Indigenous coauthor). For example, we included indicators relating to Indigenous representation in the CRE-IQI (ie, number and percentage of Indigenous researchers involved in projects; number and percentage of Indigenous authors listed on manuscripts and conference presentations and Indigenous students as a proportion of students overall), as indicators of impact.

From the outset, the evaluation was not explicitly guided by an Indigenous research paradigm, but rather by a set of guiding principles and an ‘all teach, all learn’ approach. As the evaluation progressed, there was increased scholarship on Indigenous-informed evaluation frameworks, and the evaluation team found itself adapting as best we could in a fast-evolving evaluation landscape. Primarily, however, it was through increasing Indigenous participation and leadership in the CRE-IQI overall that we could incorporate the values, priorities and perspectives of Indigenous people in all stages of the evaluation.

### A strengths-based approach that acknowledges and moves beyond practices that have harmed Indigenous peoples in the past

We reflected that employing developmental evaluation as the primary approach inherently emphasises strengths while addressing areas for improvement. Additionally, we prioritised maintaining a strengths-based narrative rather than resorting to deficit discourse in discussions and reporting. For instance, when discussing health inequalities for Indigenous Australians, we highlighted their strengths and resistance, resilience and adaptability in the face of adversity. Throughout the evaluation, we engaged in extensive discussions within the evaluation team and broader CRE-IQI collaboration, acknowledging the importance of avoiding deficit discourses.

The relationships established among Indigenous and non-Indigenous members were crucial to the evaluation. As the Indigenous co-authors noted, ‘*Everyone involved is dedicated to improving health outcomes for Aboriginal and Torres Strait Islander people. Long-term relationships have provided confidence to grow together in a strengths-based, positive way*’. However, there was room for increased Indigenous leadership and participation in determining evaluation questions and in generating and analysing data, particularly concerning the evaluation interviews.

### Translation of findings into sustainable changes in policy and/or practice

There was strong sentiment that translation was intrinsic to the evaluation given that it was a key cross-cutting programme of the CRE-IQI. Thus, translation was embedded in the design of the evaluation—a participatory design primarily intended to inform the ongoing formation and function of the CRE-IQI. As part of our reflexive dialogue, we discussed the many adaptations that had been made to the operation of the CRE-IQI because of feedback from the evaluation data, and the way that the lessons learnt from this continued to be applied.

Evaluation findings informed the thinking and implementation around a new phase of the research collaboration, which is the Indigenous-led Centre for Research Excellence in Strengthening Systems for Indigenous Healthcare Equity (CRE-STRIDE) (https://cre-stride.org). These findings are reflected, for example, in the CRE-STRIDE policy that all associated research projects are Indigenous-led/co-led, and in the continuing use of developmental evaluation and the application of an Indigenous evaluation framework^1^ in the ongoing CRE-STRIDE evaluation.

### Providing benefit to Indigenous participants

As part of the reflexive dialogue, we concluded that the evaluation benefitted Indigenous participants and health services involved in the CRE-IQI in several ways. These included the diversification of the collaborative group to include greater Indigenous leadership, the cocreation of knowledge with participants, and the facilitation of early access to evaluation findings (ie, without waiting for research publications). These benefits were enabled by the participatory nature of the evaluation. The evaluation also helped to identify priorities for the next phase of the Indigenous research collaboration—specifically the need to increase intersectoral representation to support research partnerships in addressing the social and cultural determinants of health in a holistic way.

### Strengthening evaluation capacity and providing opportunities to learn from one another

The QAT states that a research project should leave a legacy of additional skills, experience and knowledge in the participating Indigenous community. Within the evaluation, there was an emphasis on learning from one another through the implementation of our ‘all-teach, all-learn’^41^ research capacity-strengthening framework. Evaluation findings were presented to CRE-IQI Research Capacity-Strengthening seminars and we held several Masterclasses on evaluation. Both these were attended by Indigenous researchers, health service staff and community members. However, unlike several of the individual CRE-IQI research projects, no Indigenous staff were employed specifically to conduct the evaluation, nor were businesses owned by Indigenous people engaged to design any of the evaluation graphics or reports.

## Discussion

In this study, we assessed the quality of an evaluation of a national research collaboration by undertaking a reflexive dialogue, informed by the QAT, with Indigenous and non-Indigenous members of the CRE-IQI. Our analysis found examples of adherence to each QAT domain to some extent, but no domain was fully achieved to our satisfaction. For example, areas for improvement included the need to focus on engagement with, and leadership by Indigenous people in evaluations and programmes of work related to them, while strengths of the evaluation included being adaptable and responsive to emerging issues for the collaboration.

The reflexive dialogue we used involved defining our standards for each QAT domain across our two coauthor groups, and redefining these as our understandings shifted when different perspectives and aspirations were raised. For example, although Indigenous people had always played some form of leadership role in the CRE-IQI, our understanding of the diverse forms that leadership can take and an awareness of the barriers to achieving these became more sophisticated and improved over the lifespan of the research collaboration. Asking ‘how could we do this better’ is fundamental to quality improvement and CQI research and came naturally as a group of quality improvement researchers. In doing so, it only deepened our aspirations and benchmarks for greater Indigenous leadership in the future.

These conversations also raised questions about the nature of ‘quality’ and how best to think of it in this context. Quality itself is a subjective term^42^ though it has been argued that it should only be determined by the beneficiaries of the research.^3^ If we take quality to mean ‘perfection’ or even ‘exceptional’, our appraisal raises examples of where the evaluation could be improved through Indigenous leadership, control over evaluation materials and adopting an Indigenous paradigm. If, however, we take quality to mean ‘fit for purpose’, then our results strongly support the appropriateness of the developmental approach in attending to the shifting context of the CRE-IQI. Going further, quality may also mean ‘transformative’, which involves power shifting to empower participants.^42^ The CRE-IQI evaluation could be seen as transformative because it played a crucial role in the successful refunding of the CRE-IQI as CRE-STRIDE with its 50% Indigenous scholar team and an Indigenous Chief Investigator (VM). Our results also demonstrate the benefits of our process of colearning that underpinned both the evaluation and interactions across all CRE-IQI teams.

To this end, further research and development is needed both to assess the quality of evaluations of collaborations and to develop metrics that are driven by Indigenous perspectives. Vine *et al*,^18^ in their 2023 scoping review of Indigenous-specific evaluation tools, frameworks and guidelines, concluded that despite a small yet growing number of all three, reporting and utilisation of them is still very limited. However, this could be related to expected time lags, and we could see an encouraging upward trend in both reporting and use over coming years.^18^

As authors on this manuscript, we have all worked together in various capacities over many years and developed bonds and trusting relationships that are underpinned by a strong shared commitment to improving health outcomes for Indigenous communities. Overall, we found the mutual sharing of stories and perspectives that emerged through a systematic reflexive dialogue between authors a highly valuable collective learning exercise. Despite this, we found that carrying out a reflexive dialogue was challenging at times. We offer the following reflections on this process to support others in building a culture of continuous reflexivity and translating our insights into action.

First, a reflexive process with peers needs to be deliberate and slow-paced to enable deep reflection and discussions. Second, the discussions were most useful, because they gave us opportunities to explore ideas and perspectives and then have them challenged or confirmed in a safe space. Third, we established separate groups initially to mitigate against any potential power imbalances and to ensure that all perspectives were developed, presented and discussed, with the aim of generating wider group discussion and enhancing mutual understanding. This set-up also supported the creation of safe spaces for discussion. Space for, and acceptance of dissenting voices is particularly important when individuals come from different world views. For us, it supported learning and highlighted the value of this process for diverse collaborations. Finally, given our experience, we emphasise the process of reflection within teams, and the value that comes from this, as both an educative tool and a way to develop shared action.

The QAT is designed for multiple purposes including to appraise the quality of systematic reviews, journal articles and proposals of research that involve Indigenous people, and as an educative tool to support researchers in undertaking respectful, high-quality research with Indigenous people and communities.^11^ Although the QAT is well cited in the peer-review literature, we could not identify its application as a tool to guide a reflexive dialogue for assessing evaluations. Our study shows, however, that the QAT can be applied retrospectively, in the spirit of learning and improving, to guide a reflexive process on a completed evaluation of a collaboration.

The views presented in this paper are those of the coauthors, and are not necessarily held by other members of the CRE-IQI. Furthermore, because all authors were embedded within the CRE-IQI we could be viewed as having a vested interest in presenting the evaluation in a positive light. However, this process was deliberately reflexive and undertaken within a framework of wanting to strive for improvement. It reflects our desire to always be self-reflexive and do better.

## Conclusion

Although reflexive evaluation practice is not always comfortable, it does provide an opportunity to generate insights for improvement. Reflecting as we did—in a partnership between Indigenous and non-Indigenous colleagues—enabled deeper insights and meaning, as well as relationship building. We hope that our process models how other research in Indigenous contexts might better advance ethical, quality Indigenous research through foregrounding Indigenous researchers and communities.

## Supplementary material

10.1136/bmjgh-2023-014433 online supplemental file 1

10.1136/bmjgh-2023-014433 online supplemental file 2

## Data Availability

All data relevant to the study are included in the article or uploaded as supplementary information.
