# Supplementary material for: Reflecting on the quality of a methodologically pluralist evaluation of a large-scale Indigenous health research collaboration in Australia
Source: BMJ Glob Health. 2024 Aug 3;9(8):e014433. doi: 10.1136/bmjgh-2023-014433 (PMC11298732; doi:10.1136/bmjgh-2023-014433)
Supplement: online supplemental file 1 [file bmjgh-9-8-s001.pdf]

Supplementary file 1

Table 1: Shown here are the domains and their descriptions of the Quality Appraisal Tool for Aboriginal and Torres Strait Islander research, based on the work of Harfield et al.(2020).

Domain in QAT	Description of QAT domains
1. Did the research respond to a need or priority determined by the community?	For the research project to be acceptable and accountable to the Aboriginal and Torres Strait Islander community participating in the research, it must respond to the identified needs and priorities of that community. This means the research should be in response to priorities arising from, negotiated with and endorsed by, the community, thereby ensuring the project's relevance.
2. Was community consultation and engagement appropriately inclusive?	As with other communities across Australian society, Aboriginal and Torres Strait Islander communities are diverse and generalisations about community views cannot be made. To ensure good practice, it is appropriate that researchers engage with, and be inclusive of, a range of organisations, groups and governance structures within each community both prior to and throughout the research.
3. Did the research have Aboriginal and Torres Strait Islander leadership?	Any research with Aboriginal and Torres Strait Islander individuals, families and communities should have Aboriginal or Torres Strait Islander leadership to ensure Indigenous ways of knowing, being and doing are represented throughout the project. At least one Aboriginal and Torres Strait Islander person should be among the principal investigators, and/or be a member of the research leadership team.
4. Did the research have Aboriginal and Torres Strait Islander governance?	Ensuring Aboriginal and Torres Strait Islander authority over the research project means researchers must work together with the appropriate community leaders and members throughout the research process. Researchers should be guided by a relevant existing governance structure or establish one (or more) such structures that are acceptable to a community or communities. Adhering to these governance structures enables relevant cultural and contextual knowledge to inform the research.
5. Were local community protocols respected and followed?	Researchers must accept that Aboriginal and Torres Strait Islander community obligations and protocols are the top priority for community members, and that protocols can differ between communities. Unexpected events such as 'sorry business' may impact upon research timelines and outcomes as community leaders and members fulfil their obligations.

	Researchers have a responsibility to familiarise themselves with and follow local community protocols before engagement commences, including making use of interpreters as required, to reduce the risk of protocol breaches.
6. Did the researchers negotiate agreements in regards to rights of access to Aboriginal and Torres Strait Islander peoples' existing intellectual and cultural property?	A research project's rights of access to Aboriginal and Torres Strait Islander peoples' existing intellectual and cultural property should be subject to a formal agreement prior to the research commencing. A legally binding agreement is preferred over a Memoranda of Understanding as this is a better way of protecting and promoting the interests of Aboriginal and Torres Strait Islander peoples. The agreement should set out the roles and responsibilities of researchers and community members involved in the research, and clearly identify who owns the research data. It should also identify the benefits to the community as determined by the community, including resource sharing and training to be delivered as part of the research.
7. Did the researchers negotiate agreements to protect Aboriginal and Torres Strait Islander peoples' ownership of intellectual and cultural property created through the evaluation?	All published material resulting from the research project must abide by the National Health and Medical Research Council's *Values and Ethics Guidelines for Ethical Conduct in Aboriginal and Torres Strait Islander Health Research*. This stipulates that new knowledge generated through research remains the intellectual property of Aboriginal and Torres Strait Islander contributors. Researchers must also acknowledge all contributions by Aboriginal and Torres Strait Islander community members in research outputs, along with those of researchers, research participants and governance bodies.
8. Did Aboriginal and Torres Strait Islander peoples and communities have control over the collection and management of research materials?	The appropriate collection and management of all biological and non-biological research materials must remain under Aboriginal and Torres Strait Islander control. Collection of such materials must be undertaken in a respectful manner, be specific to the particular research project, and ensure that participating Aboriginal and Torres Strait Islander people remain the owners of the data they provide.
9. Was the research guided by an Aboriginal and Torres Strait Islander research paradigm?	Prior to and during the research project, researchers should be guided by an Aboriginal or Torres Strait Islander research paradigm that acknowledges Indigenous ways of knowing, being and doing, and is based on lived experience. This will ensure the research methodology reflects the values, priorities and perspectives of research participants and their communities.
10. Does the research take a strengths-based approach, acknowledging and moving beyond practices that have	Acknowledging and fostering Aboriginal and Torres Strait Islander strengths and resilience should be a core focus of researchers and research practices, breaking from historic approaches which have been harmful to communities. A key requirement is that research

harmed Aboriginal and Torres Strait Islander peoples in the past?	contributes to improved health, social and economic outcomes for Aboriginal and Torres Strait Islander communities.
11. Did the researchers plan and translate the findings into sustainable changes in policy and/or practice?	The research team must plan from the outset to disseminate its research processes and findings to relevant individuals and organisations. Good knowledge translation plans are a key ingredient in driving sustainable improvements in policy settings and/or health service delivery. Positive and lasting change is more likely when research is done in partnership with Aboriginal and Torres Strait Islander communities.
12. Did the research benefit participants and Aboriginal and Torres Strait Islander communities?	Any research project involving an Aboriginal or Torres Strait Islander community must do more than describe the issues. It must also produce meaningful benefits as determined by the community participating in the research. It is important that these benefits accrue not just to the community involved but also to Aboriginal and Torres Strait Islander peoples more broadly.
13. Did the research demonstrate capacity strengthening for Aboriginal and Torres Strait Islander individuals?	A research project should leave a legacy of additional skills, experience and knowledge in the participating Aboriginal and Torres Strait Islander community to ensure community members have greater capacity to assist with, implement and take leadership of future research. Wherever possible, community members should be trained and employed throughout a research project. Local Aboriginal and Torres Strait Islander businesses, or those employing community members, should also be prioritised for providing services to the research project. Such investments strengthen communities, improve individual health and wellbeing, and promote the advancement of all Aboriginal and Torres Strait Islander peoples.
14. Did everyone involved in the research have opportunities to learn from one another?	The research activity must provide avenues for all involved to gain key learnings from each other through a two-way learning process focused on building future capacity. Aboriginal and Torres Strait Islander individuals, families and communities should have opportunities to learn about all components of the research process. Non-Aboriginal researchers and their research communities should take the opportunity to learn from Aboriginal and Torres Strait Islander researchers and participants about their culture and ways of knowing, being and doing. At the end of the research project, researchers should allocate appropriate resources and time to present their findings back to research participants and their community.

Harfield, S., Pearson, O., Morey, K. *et al.* Assessing the quality of health research from an Indigenous perspective: the Aboriginal and Torres Strait Islander quality appraisal tool. *BMC Med Res Methodol* 20, 79 (2020). https://doi.org/10.1186/s12874-020-00959-3.
