# Supplementary material for: Reflecting on the quality of a methodologically pluralist evaluation of a large-scale Indigenous health research collaboration in Australia
Source: BMJ Glob Health. 2024 Aug 3;9(8):e014433. doi: 10.1136/bmjgh-2023-014433 (PMC11298732; doi:10.1136/bmjgh-2023-014433)
Supplement: online supplemental file 2 [file bmjgh-9-8-s002.pdf]

Supplementary file 2

Table S2.1 Indigenous and non-Indigenous perspectives on the quality of the CRE-IQI evaluation, drawing on the domains espoused in the Quality Appraisal Tool for Aboriginal and Torres Strait Islander research, based on the work of Harfield et al. (2020).

**Questions posed for as part of the reflexive dialogue, adapted from QAT.**	**Non-Indigenous co-authors' perspectives**	**Indigenous co-authors' perspectives**
**1. To what extent did the evaluation respond to a need or priority determined by the community?**	When considering this domain, we first debated who constituted the relevant 'community'. The CRE-IQI was a research collaboration comprised of many research projects rather than a specific one. Therefore, we considered its members and member organisations, as well as the communities they serve, as our community. We identified that the community voice was heard through the Aboriginal and Torres Strait Islander PHC services (which included community controlled and government-managed services) and member regional support organisations established to advocate for, and provide health services to, Aboriginal and Torres Strait Islander people.<br>We recalled how the CRE-IQI concept emerged from priorities identified by, and with, Aboriginal and Torres Strait Islander people and organisations as part of earlier collaborations focusing on CQI in Aboriginal and Torres Strait Islander PHC, and their contribution to the funding application for the CRE-IQI. As part of this process, community priorities were defined and endorsed in order to get an application for funding together.	The first question highlighted the shortcomings of utilising a project-oriented appraisal tool for a large-scale collaboration. Who was 'the Indigenous community' in the context of the CRE-IQI? We took it to be the Aboriginal and Torres Strait Islander membership of the collaboration including Indigenous researchers, policymakers and staff from Aboriginal and Torres Strait Islander health services. The evaluation was based on the aims of CRE-IQI such as the development of new tools, improving data systems, building QI capacity etc. One of the aims was to explicitly monitor and evaluate the innovation platform. We were unsure what level of input Aboriginal and Torres Strait Islander collaborators had into determining these priorities. It may have occurred implicitly, i.e., not so visible to those outside the coordination centre and CRE-IQI proposal development (consultation must have been demonstrated in the proposal). At the time, CQI was a high priority for Aboriginal and Torres Strait Islander health services with the release of the CQI National Framework.

	We discussed how the evaluation of the CRE-IQI was one of the five research aims defined in the resulting funding proposal to the NHMRC, and that it included a funding allocation and commitment to using multiple evaluation approaches. We reflected on the reasons why we wanted multiple approaches including: the need to be accountable to the community, to understand how the collaboration operated, and how to use an interactive method to respond to complexity and emerging issues identified by the community. As a group we also reflected that at the time we established the evaluation, we explored different evaluation approaches to ensure they would be 'fit for purpose' within the context of the CRE-IQI. Fit for purpose was important as the design would enable us to adapt to emerging priorities, it was not an evaluation we just did at the end to consider the outcomes.<br>Another area we discussed was how the development of the evaluation priorities over time allowed them to be reflexive and responsive to emerging issues identified by CRE-IQI members at regular meetings.	
**2. To what extent was the community consultation and engagement appropriately inclusive?**	Our concept of 'community' for this domain were the CRE-IQI members, who, as a group, represented multiple organisations and roles. Thus, our community also incorporated the many Aboriginal and Torres Strait Islander stakeholders and organisations that had been closely involved in discussions about the evaluation priorities. We discussed how a number of the outputs from the evaluation were a direct result of feedback from our Aboriginal and Torres Strait Islander members, and	Similarly, we're not sure how consultation occurred for the evaluation. There was one Aboriginal person on the Evaluation Working Group. We recognise that the CRE-IQI had an Indigenous advisory group, but the effectiveness of this structure was limited by people's availability. Some consultation occurred during face-to-face meetings. There was limited Aboriginal and Torres Strait Islander membership in the collaboration early on but this grew over time. We thought the evaluation design (FAIT, Network Analysis, DE) was fairly pre-set and it was unclear the level

	how purposeful the evaluation was in responding to data and issues arising over the duration of the CRE-IQI.	of Indigenous collaborator input in the design. There was stronger engagement with the collaboration for the developmental evaluation aspect, allowing for input along the way, however clearer reporting on outcomes of the developmental changes would have made this engagement more explicit. We recognise that this is difficult to do consistently and takes time and resources. Without knowing what was going on within the EWG and/or how face-to-face sessions were prepared, reasons behind selection of evaluation methodologies beyond the major ones, e.g., 'principles-focused' evaluation were unclear. Perhaps this related to the 'emergent' design and developmental evaluation as key methodology choices driven by findings, but this pathway was not well communicated beyond the EWG.
3. **To what extent did the evaluation have Aboriginal and Torres Strait Islander leadership?**	The CRE-IQI was guided by a set of overarching principles, several of which advocated for Aboriginal and Torres Strait Islander leadership and direction in all stages of the research and evaluation processes. We discussed how these principles played out in the evaluation, noting that there were two Aboriginal investigators on the CRE-IQI investigator team.<br>We also talked about how, over time, there was an increased expectation and emphasis from the broader CRE-IQI membership on increasing Aboriginal and Torres Strait Islander leadership and participation in all aspects of the collaboration, including the evaluation. Thus, when we set up the Evaluation Working Group, we approached an Aboriginal Chief Investigator of the CRE-IQI to Chair it. However, all other participating members of the Evaluation Working Group were non-Indigenous.	The lack of Aboriginal and Torres Strait Islander leadership early in CRE-IQI has been documented within the evaluation. We believe the intent of doing Aboriginal and Torres Strait Islander research the right way (and by extension its evaluation) was there as reflected in the CRE's principles and value statements, however, the collaboration did not have enough Aboriginal and Torres Strait Islander members or leaders actively involved for the first couple of years. During this time Aboriginal and Torres Strait Islander people involved in the broader collaboration felt respected, but not necessarily listened to or engaged. '*If you're a lone ranger in that space, don't have enough back-up, it is hard to shift*.' In these situations, you feel the weight of being the lone voice, feeling you had little influence and your voice is lost.

	This was due to the researchers having the particular evaluation methodological expertise required.<br>The discussions we had as part of the Evaluation Working Group (and more broadly) included purposefully increasing participation by Aboriginal and Torres Strait Islander researchers and stakeholders in all stages of the evaluation's design, data collection, and interpretation and analysis of the data. We recalled the CRE-IQI's efforts on increasing the representation of Aboriginal and Torres Strait Islander stakeholders and organisations at biannual meetings, where evaluation plans and interim analysis were workshopped. In addition, we identified the additional time and energy spent on gathering Aboriginal and Torres Strait Islander perspectives when collecting evaluation data as part of this domain.<br>In spite of these efforts, and the challenges we faced given our time constraints and context, we had a strong sense that more could have been done in this area. One challenge we discussed was how to share and facilitate Aboriginal and Torres Strait Islander leadership and participation when there are so many competing demands on our First Nations colleagues, both at work and in their private lives. To give one example, we initially had an additional Aboriginal member in the Evaluation Working Group but unfortunately, due to other work commitments, they were unable to continue their involvement.	Due to low numbers, the EWG had one Aboriginal and Torres Strait Islander evaluator over the course of the CRE-IQI evaluation.<br>We spoke about what Aboriginal and Torres Strait Islander leadership looks like, what does it mean as it is a new experience for some. It is not just about leading a project, it is about having influence, and having contributions respected and discussed within an 'all teach, all learn' framework.<br>For future consideration, we spoke about the need to document over time how and where Aboriginal and Torres Strait Islander people were involved, from the beginning during ABCD Research Partnership at the health services through to the start of CRE-STRIDE. This would capture the contributions of Aboriginal and Torres Strait Islander people who may not have had the research qualifications, but who still contributed their valuable skills and experience. We did capture some of that in the CRE-IQI Network Analysis, but it was only part of the picture and provides limited context to their story. In sum, collaboration made space to listen to Aboriginal leadership during evaluation but had limited influence.
4. **To what extent did the evaluation**	We considered that oversight of our evaluation was embedded within the existing governance structures of the CRE-IQI, which included Aboriginal and Torres	As discussed, there was little Indigenous governance of the CRE-IQI evaluation. The Advisory Group did not operate and there was one Aboriginal person on the EWG.

have Aboriginal and Torres Strait Islander governance?	Strait Islander organisations established to support their community (such as Aboriginal Community Controlled Health Organisations). Initially, the CRE-IQI governance structure had included, and set up, an Indigenous advisory committee, but this committee was short-lived due to its members other onerous work commitments (as per Justification 3). The committee also felt that existing governance structures were adequate as they had strong Aboriginal and Torres Strait Islander representation.<br>The management committee of the CRE-IQI, which included Aboriginal and Torres Strait Islander stakeholders, routinely discussed the evaluation and set directions. We also found face-to-face biannual meetings of the CRE-IQI members important in terms of obtaining feedback and direction for the evaluation, and through these collaborative processes we were able to set evaluation priorities.<br>We note again that the Evaluation Working Group was chaired by an Aboriginal Chief Investigator.	
**5. To what extent were local community protocols respected and followed?**	As we reflected on this domain, we reflected on our deliberate strategy to respect the competing demands on Aboriginal and Torres Strait Islander organisations and stakeholders' time. We discussed how evaluation input and feedback were incorporated into the biannual meetings where members came together, and our specific strategy of setting interview times that were most convenient for participants, including Aboriginal and Torres Strait Islander participants. As part of the informed consent process for interviews we ensured that participants were aware of the time commitment involved in contributing to the evaluation, and that their	Another difficult question to apply to CRE-IQI evaluation. We translated this question to the use of CRE-IQI principles and values and the 'all-teach, all-learn' approach as the protocols guiding the evaluation. Developmental evaluation methodology embodies an all-teach, all-learn approach. We built on these principles and protocols throughout evaluation. We did reflect that the Aboriginal and Torres Strait Islander mentorship contribution to the 'all-teach, all-learn' framework was perhaps not as active which may have contributed to slower development of partnership and co-leadership arrangements between Aboriginal and Torres

	participation was voluntary with no disadvantage should they decline.	Strait Islander and non-Indigenous members of the collaboration.
6. **To what extent did the evaluators negotiate agreements in regard to rights of access to Aboriginal and Torres Strait Islander peoples' <u>existing</u> intellectual and cultural property?**	We reflected on our original NHMRC research proposal for the CRE-IQI, which included due diligence grant submission processes that required letters of consent/agreement from the institutions of all the Chief Investigators. Again, we talked about how the overall methodology for the evaluation was a key part of the original proposal, which had been co-designed with Aboriginal members and/or representatives of organisations serving Aboriginal people.<br>Our discussions also noted that ethics approvals for the evaluative activities were sought from two human research ethics committees – the University of Sydney, and the Northern Territory Department of Health and Menzies School of Health Research – with a specific review by an Indigenous ethics sub-committee conducted by the latter. In considering this domain we could not identify any sensitive cultural knowledge exchanged as part of the evaluation.	Questions 6 and 7 on the negotiation of agreements relate more to individual projects rather than an evaluation of a large research collaboration. There were service agreements in ABCD research however, and we reflected on the use of health service data and what we did not do so well is how we represented the data – presenting results too academically rather than from a health service perspective. There was limited discussion about data sovereignty. We spoke about entering research agreements with services, but universities make it difficult assigning IP to others and you can't necessarily do what you want to do in negotiations. How can we influence that moving forward? Model agreements for how to use data from other studies where it has been done well, like the Mayi Kuwayu study that provides a whole business model behind data sovereignty with clear Aboriginal and Torres Strait Islander governance. Through reporting requirements, health services give bucket loads of data to government, but no-one helps services use it for their own benefit.
7. **To what extent did the evaluators negotiate agreements to protect Aboriginal and Torres Strait Islander peoples'**	Our discussions for this domain centred on the importance we placed on ensuring all evaluation outputs acknowledged the contributions of Aboriginal and Torres Strait Islander people, evaluators and participants in the generation of new knowledge. For example, we recalled how Aboriginal and/or Torres Strait Islander authorship was mandated on all evaluation outputs, and in all stages of the evaluation process, in response to feedback about the need to formalise this. We also discussed our requirement that author information on peer-review	See Q6.

**ownership of intellectual and cultural property <u>created</u> through the evaluation?**	manuscripts included statements of the authors' positionality, including their First Nations status. Both of these were identified as examples of how the CRE-IQI continuously sought to improve its commitment to Aboriginal and Torres Strait Islander engagement.<br>We reflected on how the evaluation was primarily concerned with collecting and using data for the purpose of refining the ongoing operation of the CRE-IQI, and importantly that this knowledge was collaboratively created. We also considered how jointly created evaluative data and information were shared through the presentation of early findings to the CRE-IQI, member checking and participatory analysis processes. While we did not identify any specific agreements related to intellectual property, we did agree on the importance of having ethics approval and informed consent processes in place. Given that the nature of the evaluation research was collaborative, and included many organisations and people, the ownership was dispersed. We could not identify any examples in which sensitive cultural knowledge was exchanged.	
**8. To what extent did Aboriginal and Torres Strait Islander peoples and communities have control over the collection and management**	The management and storage of data collected as part of the evaluation was dictated by the University of Sydney standards and policy, which require data to be stored in secure locations and be archived and destroyed within a specified time after the evaluation. We outlined these processes in the participant information sheet, and as part of the consent processes specific to the evaluation. These processes were also included in ethics committee approvals. Again, our research was conducted in the context of the CRE-IQI governance structures in which	Aboriginal and Torres Strait Islander people participated in the evaluation but had very limited control over collection and management. As mentioned, Aboriginal and Torres Strait Islander leadership within the CRE-IQI increased towards the end of its term. We need to document this story, learn from the past and change for the future. The network analysis was a form of this documentation, but lacked a narrative component, the story that sits behind the growth of the collaboration. Acknowledge that it is difficult putting pieces together for new people because of the

**of evaluation materials?**	Aboriginal stakeholders participated. Given our deliberate inclusion of Aboriginal and Torres Strait Islander researchers and organisations in all analysis and interpretation sessions, we therefore considered the evaluation to be highly participatory. We also recollected that Aboriginal and Torres Strait Islander stakeholders were included in all discussions on how the information was to be disseminated and used to inform the ongoing operation of the CRE-IQI, as well as decisions about the merit of its work.	collaboration's long history. Need story book to tell the history.
9. **To what extent was the evaluation guided by an Aboriginal and Torres Strait Islander research paradigm?**	Although we have scored 'yes' for this domain, we acknowledge that it was only as the members reflected and learnt more over time that we felt able to describe the evaluation as being guided by an Aboriginal and Torres Strait Islander research paradigm. At the outset, the evaluation did not explicitly state that we were developing an Aboriginal and Torres Strait Islander evaluation mode. Rather, it began with a set of guiding principles, including taking a strengths-based approach, ensuring we were contextually responsive, and implementing systems, relational approaches and an emergent, interactive design. We reflected on how, over time, there had been so many conversations as a collaboration about what an Aboriginal and Torres Strait Islander way of working would be and how it would look.<br>However, we did reflect that in hindsight we could have been clearer about being guided by an Aboriginal and Torres Strait Islander paradigm from the outset. We had much discussion about how, as the evaluation progressed over several years, there was greater reflection and	Most of the methodology came from Western paradigms. For example, the FAIT impact evaluation is based on western theoretical frameworks. This was challenging when applying to the Aboriginal and Torres Strait Islander context. There was a clear need to incorporate values and meaning for Aboriginal and Torres Strait Islander people into measurement indicators. If this doesn't occur, the evaluation won't be relevant. For example, built in economic assumptions, not relevant to community context. Has to be done well and meaningful in context so that makes sense for our mob.

	increased scholarship generally on Aboriginal and Torres Strait Islander-informed evaluation frameworks. We reflected further that, in our fast-evolving research landscape, perhaps we were adapting as best we could to keep pace with these emergent conceptual frameworks. We also felt somewhat unsure about how to appropriately apply an Aboriginal and Torres Strait Islander research paradigm without Indigenous leadership specifically on the evaluation team, despite having a Chair who was Indigenous.	
10. **To what extent does the evaluation take a strengths-based approach, acknowledging and moving beyond practices that have harmed Aboriginal and Torres Strait Islander peoples in the past?**	Our discussions here covered a number of areas including how we paid specific attention to ensuring a strengths-based approach in our publications and reports. We agreed this could be evidenced through how we described health inequalities for Aboriginal and Torres Strait Islander Australians – by emphasising the great resilience and contribution of First Nations people rather than focusing on the pervasive legacy of colonisation encompassing land dispossession, displacement, disempowerment, social and economic exclusion, and ongoing racism. We reflected on the many discussions and team recognition about deficit discourses and the need to avoid these.<br>Further, the CRE-IQI, and therefore the evaluation, was guided by a set of overarching principles, the first of which related to respecting the past and present experiences of Aboriginal and Torres Strait Islander people. Importantly we saw this principle being implemented through the evaluation team ensuring Aboriginal and Torres Strait Islander input and feedback into the data collection, analysis and interpretation.	Using Developmental Evaluation as the principal approach is inherently strengths-based, analysing aspects of the collaboration to improve on, such as Aboriginal and Torres Strait Islander co-leadership and modifying methodologies such as FAIT to increase relevancy to Aboriginal and Torres Strait Islander people. Relationships that have been developed in this collaboration over long time are critical. Everyone involved is dedicated to improving health outcomes for Aboriginal and Torres Strait Islander people. Long-term relationships have provided confidence to grow together in a strengths-based, positive way. However, some deficit research practices remain. For instance, evaluation questions largely determined by non-Indigenous researchers, data generated and analysed by non-Indigenous researchers etc. But perhaps most importantly, the numbers of Aboriginal and Torres Strait Islander participants in interviews, social network analysis etc. was low – this means that results provide a biased view. The significance of this - when you work toward particular outcomes for a particular group but that group's voice is quiet you work

		from a different starting point, create different strategies and head in a direction that might not benefit that group.
11. **To what extent did the evaluators plan and translate the findings into sustainable changes in policy and/or practice?**	We felt strongly that as a key cross-cutting program of the CRE-IQI, translation was intrinsic to the evaluation. This was evident in the design of the evaluation itself – a participatory design primarily intended to inform the ongoing formation and function of the CRE-IQI. We discussed the many adaptations made to the operation of the CRE-IQI as a result of evaluative data. We also discussed how the findings from the evaluation informed the thinking and implementation around a new phase of work after the CRE-IQI – the Aboriginal and Torres Strait Islander-led CRE-STRIDE. We further reflected on how the evaluation approach in the CRE-IQI has informed the way the evaluation of the CRE-STRIDE was conceptualised and is now being implemented.	From early ABCD days, the CQI approach remains a key feature operating within the Aboriginal and Torres Strait Islander primary health care sector. At a broad-level, learnings from the CRE-IQI led to the successful funding CRE-STRIDE. Most of the evaluation has been written up within reports and academic papers. Translation to health services and community has occurred at a project-level. Sustainability question difficult to answer as many CRE-IQI projects are still operating, for example, Lessons from the Best that became Leveraging Effective Ambulatory Practice (LEAP), the learning from which led to successful Partnership project application to develop local co-designed workforce models.
12. **To what extent did the evaluation benefit participants and Aboriginal and Torres Strait Islander communities?**	In thinking about this domain we reflected on how the evaluation was designed to inform the foundation and functioning of the CRE-IQI. The participatory nature of the evaluation enabled early access to new knowledge and the opportunity to co-create knowledge with CRE-IQI members. This in turn enabled members to access information early without waiting until a research publication was released. However, given the scope and focus of the evaluation was on the CRE-IQI rather than a specific research project, there was no clear way to define specific benefits to participants and communities. As discussed above in Justification 11, our evaluation findings did inform our approach to the CRE-STRIDE as being Aboriginal and Torres Strait Islander led, an initiative that will benefit communities.	The evaluation benefitted Indigenous participants of the CRE-IQI. It has now diversified to include greater Aboriginal and Torres Strait Islander leadership. There is still more work to be done to increase intersectoral representation that will enable partnerships to address social and cultural determinants that will hopefully beneficially impact Aboriginal and Torres Strait Islander communities. Also see Question 10.

**13. To what extent did the evaluation demonstrate capacity strengthening for Aboriginal and Torres Strait Islander individuals?**	As the evaluation findings were presented to our Research Capacity Building seminars and we held a number of Masterclasses on evaluation, both of which included Aboriginal and Torres Strait Islander members, – by their very nature this would have built some capacity. In addition, by participating in biannual meetings and governance structures, these members would have been exposed to evaluative activities and, again, had some level of capacity building. There was a particular emphasis on mutual learning and the development of a strategy "All teach, all learn". However, we decided on 'no' for this domain as there was no employment of Aboriginal or Torres Strait Islander people as part of the evaluation, nor did we use local businesses owned by or employing Aboriginal or Torres Strait Islander people to provide services, such as the design of graphics and report layouts for research outputs.	There was some capacity development for the Aboriginal evaluator on the EWG, e.g., learning about social network analysis. Apart from this, the evaluation did not involve Aboriginal and Torres Strait Islander individuals. The situation might have been constrained by not having a dedicated person in a long-term position to facilitate capacity-strengthening in this way.
**14. To what extent did everyone involved in the evaluation have opportunities to learn from one another?**	We landed on a 'yes' for this domain as it was evident that the evaluation was participatory with high value placed on learning and interacting to inform the operations of the CRE-IQI. We also discussed our (the team and the CRE-IQI) overarching motto of 'All teach, all learn', which was collaboratively developed to embody the value placed on mutual learning between all involved in the CRE-IQI and in its evaluation.	Learnings occurred for those within the EWG and participants at face-to-face meetings where the evaluation was discussed and workshopped.

Harfield, S., Pearson, O., Morey, K. *et al.* Assessing the quality of health research from an Indigenous perspective: the Aboriginal and Torres Strait Islander quality appraisal tool. *BMC Med Res Methodol* 20, 79 (2020). https://doi.org/10.1186/s12874-020-00959-3
